# The spread of a wild plant pathogen is driven by the road network

**DOI:** 10.1371/journal.pcbi.1007703

**Published:** 2020-03-31

**Authors:** Elina Numminen, Anna-Liisa Laine

**Affiliations:** 1 Research Centre for Ecological Change, University of Helsinki, Helsinki, Finland; 2 Department of Mathematics and Statistics, University of Helsinki, Helsinki, Finland; 3 Department of Evolutionary Biology and Environmental Studies, University of Zurich, Zurich, Switzerland; BioSP, INRA, FRANCE

## Abstract

Spatial analyses of pathogen occurrence in their natural surroundings entail unique opportunities for assessing in vivo drivers of disease epidemiology. Such studies are however confronted by the complexity of the landscape driving epidemic spread and disease persistence. Since relevant information on how the landscape influences epidemiological dynamics is rarely available, simple spatial models of spread are often used. In the current study we demonstrate both how more complex transmission pathways could be incorpoted to epidemiological analyses and how this can offer novel insights into understanding disease spread across the landscape. Our study is focused on *Podosphaera plantaginis*, a powdery mildew pathogen that transmits from one host plant to another by wind-dispersed spores. Its host populations often reside next to roads and thus we hypothesize that the road network influences the epidemiology of *P. plantaginis*. To analyse the impact of roads on the transmission dynamics, we consider a spatial dataset on the presence-absence records on the pathogen collected from a fragmented landscape of host populations. Using both mechanistic transmission modeling and statistical modeling with road-network summary statistics as predictors, we conclude the evident role of the road network in the progression of the epidemics: a phenomena which is manifested both in the enhanced transmission along the roads and in infections typically occurring at the central hub locations of the road network. We also demonstrate how the road network affects the spread of the pathogen using simulations. Jointly our results highlight how human alteration of natural landscapes may increase disease spread.

## Author summary

Studying pathogen transmission dynamics within their natural environments can yield important new insights both on the known and unknown determinants of the real-world transmission process. In this study we analyse how a fungal plant pathogen occurs within a landscape, showing that the road network dictates where the pathogen occurs, not only by providing suitable habitat for the host plant, but also by enhancing transmissions along the roads. Mechanistic understanding of how and where the transmission is expected to occur can yield novel insights into the ecology of pathogens, and is essential for design of control strategies.

## Introduction

The process of transmission is a critical component in understanding the ecology of any pathogen. It is driven both by the within-host processes, that influence the transmissability of the pathogen in various ways [1], as well as the between-host processes that jointly determine the potential targets of transmission. The realized transmission pathway, i.e. the progression of infection from one host to another, thus often exhibits distinctive patterns, as the host type or spatial position can critically influence its probability of getting infected and the most likely sources of infection. For example, one often observed pattern across different pathosystems is the existence of *superspreaders*, where relatively few hosts are responsible for a disproportionally large fraction of new transmissions and thus pathogen persistence [2]. Such patterns could arise due to the dissimilar within-host processes leading to variation in infectiousness, but also due to between-host processes governing the amount of potentially infectious contacts. This could be the case when hosts vary in their transmission potential, or when the environment, e.g. the climate, enhances or suppresses transmissions [3, 4]. Hosts could also have different amounts of infectious contacts, due to location or behavior, leading to the same phenomena [5].

The challenge for epidemiological studies is that information on infectious contacts and transmission success rarely exists. When hosts are mobile and lead complicated lives, even the task of outlining the relevant elements involved could be challenging. Recently a substantial amount of methodological work has been dedicated to reconstructing the transmission pathways from different kinds of epidemiological data, including for example genetic information of the sampled pathogens [6, 7]. In addition to unravelling the course of events, when combined with other information, such approaches can reveal interesting properties in transmission pathways, such as the spatial extent of spread or typical characteristics of the transmission recipients and donors.

In general, a natural assumption is that the movement of pathogens and hosts and the intensity and amount of contacts between them always plays a role and should be incorporated into epidemiological analyses. For example, studies have shown the significant impact of the social network among giraffes coinciding with the patterns of direct transmission of *E.coli* among them [8], the global air-traffic volumes to be an important factor explaining the pandemic spread of influenza strains [9], and the road networks to explain the prevalence of measles cases in Niger [10] and the spread of rabies in Tanzanian dogs [11]. Spatial epidemiological analyses have utilized a diverse set of modeling tools, spanning from lattice, diffusion and metapopulation models to network models [12]. However, from all the different types of spatial epidemiological models, it appears that network models may be best suited for the analysis of highly heterogenous systems [13]. A convincing body of theoretical evidence demonstrates how predicted epidemics on networks with heterogenous features exhibit nuanced features [14] and deviate from our baseline predictions, for instance predicting high impact of the initial location of the epidemic on its success [15].

In this study we assess the effect of the road network on the transmission of a wild plant pathogen within a natural archipelago system, inside of which the almost 4000 host populations are scattered in a fragmented manner. The landscape is strongly influenced by humans, especially due to agricultural practices, with the road network effectively spanning over the entire populated area. Ecological impacts of roads are diverse: by fragmenting the landscape they influence the movement and dispersal of many other species besides the humans [16, 17]. Roadsides themselves induce a unique environment which undergoes a constant stress induced by traffic, and exhibits a distinctive spatial topology. Our study is focused on *Podosphaera plantaginis*, a powdery mildew fungal pathogen, which transmits from one host plant to another with wind-dispersing spores. Moreover, its host plant *Plantago lanceolata* is a pioneer species that often grows along roadsides, where competition between species is minimal due to regular mowing. In previous analyses of pathogen population dynamics the presence of a road within a local host population was found to have a positive effect on the pathogen population presence, but the mechanisms and implications of this finding have not been further assessed [18]. We present two alternative ways to explicitly incorporate the road network into a statistical model for the transmission dynamics of *P. plantaginis*. First, we fitted an explicit transmission model to presence-absence time-series of the pathogen, allowing us to estimate the dispersal distances and rates along the road and along the land. Second, using statistical modeling, we show that pathogen populations are most likely to establish in the central locations of the road network.

Our study demonstrates how information on a complicated transmission network can be incorporated into analyses to better understand disease spread. We anticipate that adopting a similar network perspective could improve our understanding of a broad range of spatially structured biological systems. Regardless of the network type, e.g. a road- or a river network, its properties can induce dispersal routes, as well as unique habitats alongside to them that influence disease transmission. In particular, this approach could be essential for the sustainable management of plant diseases in agriculture [19], a question of major economic importance. Indeed, a range of pathosystems suggest that the underlying network (road or other) enhances disease spread: roads seem to promote the spread of the fatal root disease of Port Orford cedars [20], the spread of the poplar rust fungus occurs downstream a river network [21] and the spread of fungal diseases and invasive plants coincides with hiking- and biking trails [22]. Moreover, developing realistic dispersal models that can incorporate meteorological and anthropomorphic drivers were considered as one of key challenges in modeling plant diseases in a recent review [23]. Overall, correctly identifying the dispersal routes has the potential to provide insights into the key mechanisms driving pathogen spread and persistence, as well as insights into how genetic diversity is spatially distributed.

## Materials and methods

### Host and pathogen species

**Fig 1:**
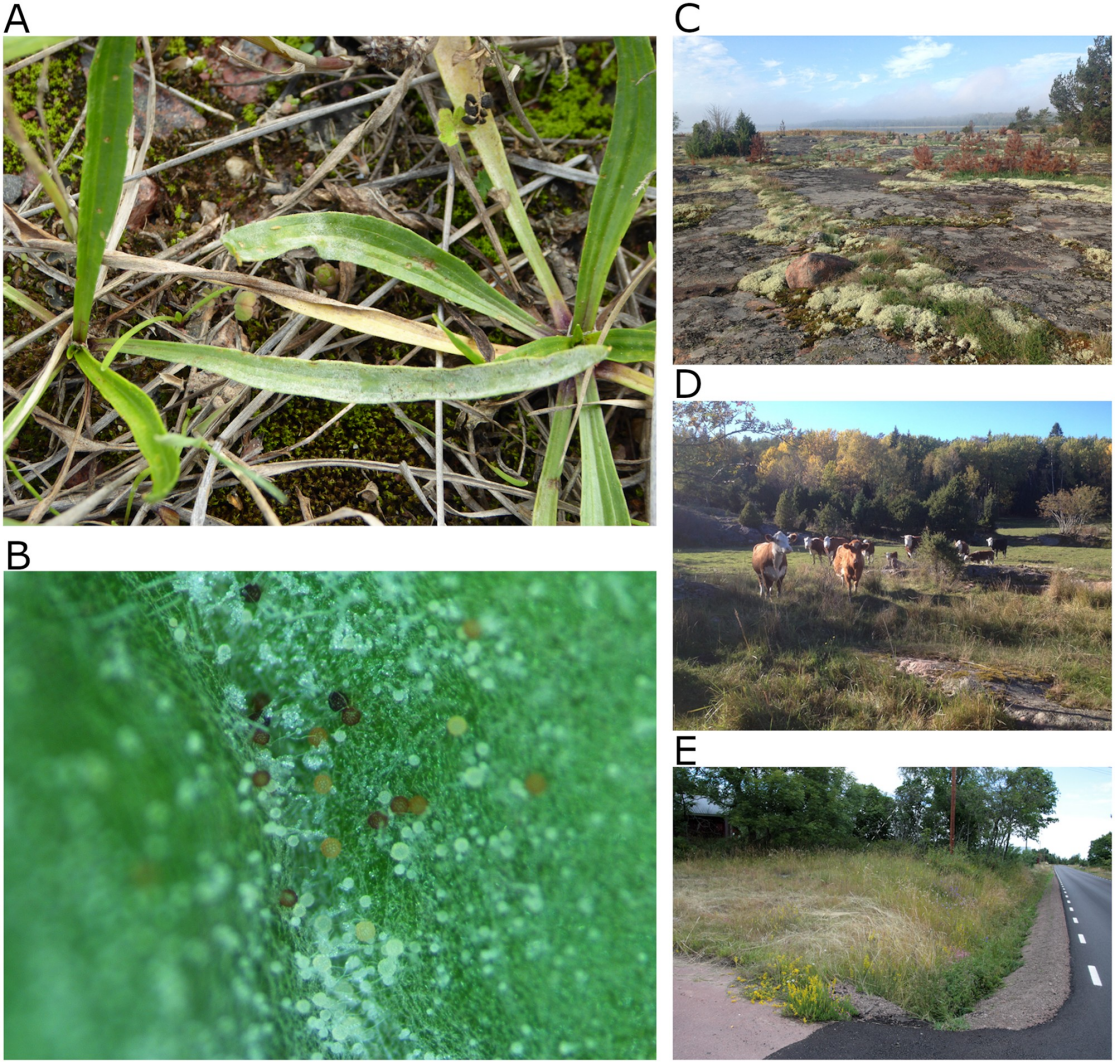
Panel A shows the powdery mildew infection on *P. lanceolata*, with a zoom-in in panel B, depicting both the clonal conidial spores that spread during the summer, and the larger and mainly darker spore structures, chasmothecia, that ensure the pathogen overwintering. Panels C-E depict example locations where the host grows in Åland archipelago.

Our study is focused on a powdery mildew *Podosphaera plantaginis*, which is an obligate, host-specific fungal biotroph infecting the ribwort plantain *Plantago lanceolata*. *Plantago lanceolata*, in turn, is a common weed of cultivated land, growing in meadows, roadsides, courtyards and coastal areas. The life cycle of the pathogen consists of a clonal epidemic phase in the summer, during which the wind-dispersed spores spread the infection. Due to the fast progression of infection, several infection generations are possible during the summer. Towards the autumn sexually-produced overwintering spores, chasmothecia, are produced. Those chasmothecia that overwinter successfully restart the epidemic again the following spring. In Finland the host and the pathogen only occur in Åland archipelago. There is considerable strain diversity in this pathosystem, and the strains vary in their life-history strategies, some producing more abundant infections and spreading faster [24]. Co-infections of pathogen strains are common, and are associated with more severe infections and enhanced local epidemics [25]. In panel A of Fig 1 we show two infected host plants, where the white spores, as well as the dark chasmothecia are both visible to eye. In panel B we show the two types of spores produced by the fungus, with the white small ones corresponding to the spores that are carried by the wind and drive the epidemics during the summer, while the dark ones are the overwintering structures. In panels C, D and E we show examples of typical habitats where the host plant grows in the Åland archipelago.

### Dispersal of fungal spores

Dispersal process of a fungal spore involves the following three phases: 1) the take-off, where the spores escape to the atmospheric layer, 2) the transport, where the spores travel in it, and 3) the deposition, where the spores land back to the surface [26]. The take-off mechanism for *P. plantaginis* is passive, involving strong enough gusts of wind. The turbulence induced by wind allows the spores to escape the quasi-laminar layer, that is close to the surface of the ground and where not much wind is present, to enter the upper layers atmospheric boundary layer, where they can travel long distances with winds. Greater release height usually leads to greater wind velocity [27]. While *P. plantaginis* spores are relatively large (conidia; 25–38 × 15–20 μm) [28], which causes them to have a considerable deposition velocity back towards the surface, simulations of spore trajectories in the air suggest that a substantial propotion of spores of even this size could travel more than 1km distance before landing back, with this average distance increasing as a function of the release height [29].

As the winds and small-scale gusts are complex and chaotic and thus challenging to model, and the fungal spore size of *P. plantaginis* falls into a size-category, in which the aerodynamics of the particles are not yet well understood, we are faced with a challenge in modeling the spore dispersal. While in wind-tunnel experiments baseline knowledge on the aerodynamics of the spores are obtained [28], such experiment cannot mimic the complicated nature of winds in the wild. On the other hand, inference on dispersal from observational data is haphazard and always tied to the spatial scale of the observations [30]. Here, we hypothesize that the road network could alter the transmission processes significantly due to the increased turbulence caused by the traffic, that enhances the take-off of the pathogen spores. In addition, since the roadsides are mowed, the canopy of the crops within them is short, and this leads to the atmospheric layer with greater wind speed to reside closer to the ground and thus also closer to the infected plants and spores [27].

### The study system

**Fig 2:**
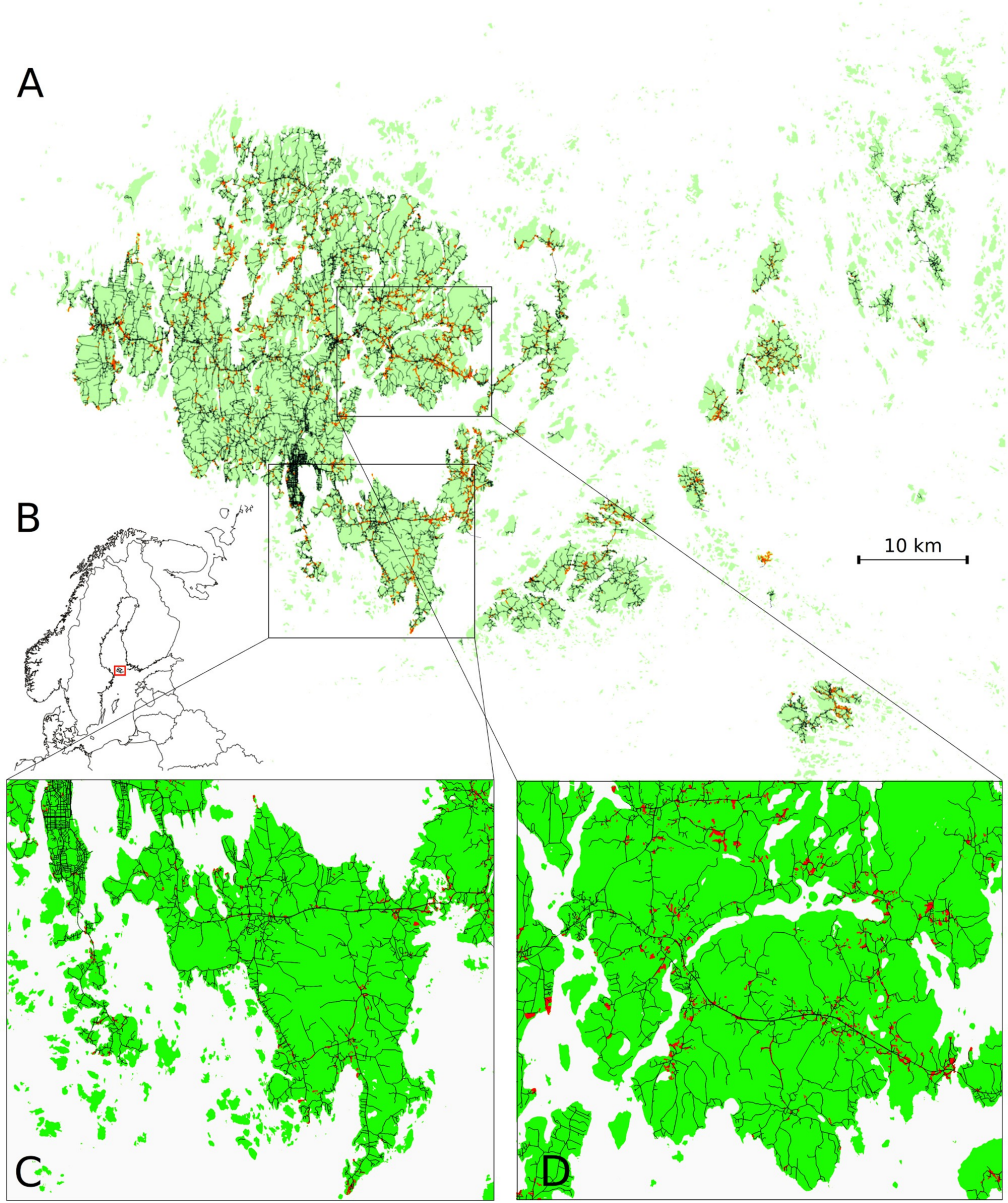
The study system consists of 3448 host populations situated in the main island of Åland island archipelago (panel A), that resides between Sweden and Finland in the Baltic sea (B). In panels A and C&D, the road network is shown in black and the host populations in red. The road network covers the main islands densely, and ferries operate between the islands. Panels C and D illustrate that typically host populations reside directly at a roadside, or in the close proximity to it. The map in panel B was produced using Geodata from European Commission, EuroGeographics for the administrative boundaries, while the other maps were produced with data produced by National Land Survey of Finland.

**Fig 3:**
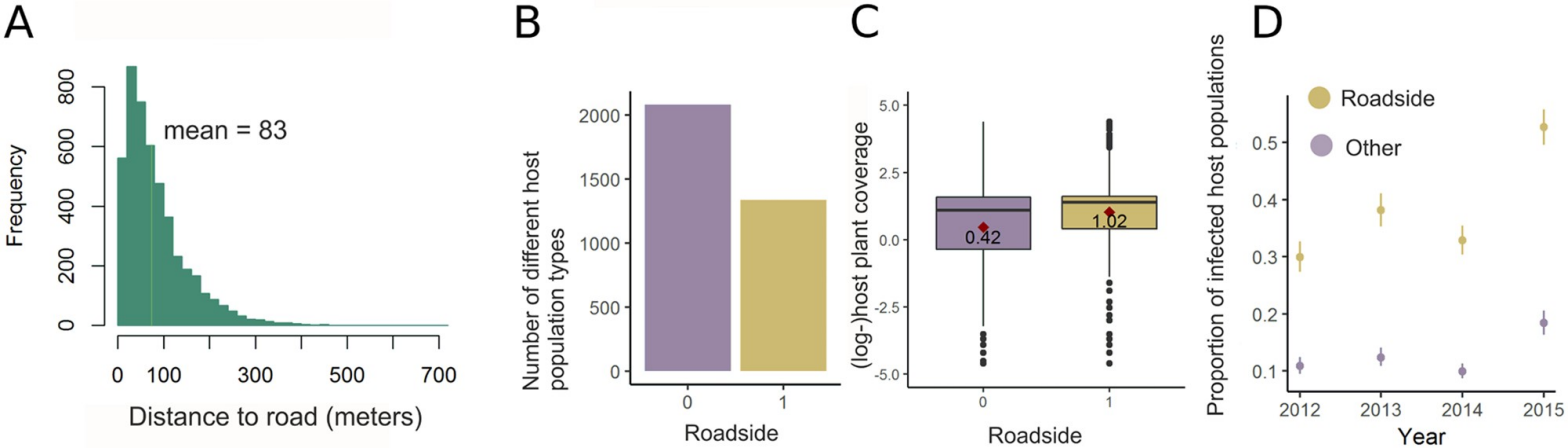
Panel A shows the distribution of distance to road from the gravitational center of the host population, B shows the total counts of roadside and non-roadside populations. C depicts the larger host plant coverages in roadside populations than the other populations and D shows the different infection prevalence between the two types of host populations.

The study system, illustrated in Fig 2, is located in Åland islands archipelago, in the Baltic sea between Finland and Sweden. It is approximately 50 x 70 kilometers in size, and in this study area, all the host plant populations were systematically and thoroughly mapped in the early 1990’s using topographic maps and then visiting all potential habitats for *P. lanceolata* [31]. While the exact number varies slightly from year to year, approximately 3448 distinct locations reside within the main island and approximately 4248 within the largest main islands. The host populations occur within the landscape in a highly fragmented manner, but are situated mostly in close proximity to the roads, as othervise the landscape is dominated by fields and forests, both constituting an unsuitable habitat for *Plantago lanceolata*. The host populations vary in their combined host plant coverage (spanning from 0.0001 to 80.35 square meters). The mean distance to a road from the (gravitational) centre of the host population is only 83 meters, and distances larger than 300 meters are rare, as seen from the histogram in Fig 3 panel A. In panel B of Fig 3 we show that 39% of the host populations were located directly at a roadside, which we defined to correspond to the case where the spatial polygon defining the borders of the host population intersects with a road. The roadside host populations on average have more host plants growing within them, as seen in Fig 3 panel C and the difference between the mean log-host coverages between the two groups is significant (*t*(12424) = −20.301, p-value < 2.2*e* − 16). During the time-span of our study (2012-2015) the number of infected populations remained around same levels, the lowest infection indicidence being 514 in year 2012 and the highest 730 in year 2015. Regarding infections, direct roadsides were more often infected than the rest of the host populations, as seen from panels D in Fig 3. An extensive study on the environmental drivers of the transmission dynamics of the *P. plantaginis* is presented in [18].

### Pathogen presence-absence data

At the beginning of September, each year during the consecutive years 2012-2015, all the host populations within the system were surveyed for the presence of infection. This was done by visual inspection, as towards the late summer the powdery mildew infections are visible to the naked eye (see Fig 1). The abundance of infection was categorized for each infected host population as follows: 0 = no infection, 1 = 1-9 infected plants, 2 = 10-99 infected plants, 3 = 100-999 and 4 = 1000 or more. Also similar measure for *relative abundance of infection* was collected. However, in all the subsequent analyses, we utilize the absolute abundance measure, as this corresponds directly to the size of the pathogen population, which can be assumed to coincide both with the force of infection it may cause, and with the amount of pathogen spores available for overwintering, and thus has a more direct mechanistic interpretation. Yet due to the small number of category 4 infections, categories 3 and 4 were merged in our statistical analyses. Regardless of the infection status of the host population, the combined host plant coverage (in square meters) within the local host population was characterized with visual inspection and recorded to the data. The survey effort, i.e. the time the surveyors spend in each local population searching for infections, is scaled relative to the geographic area of the population, and thus while there is a small probability that the surveyors fail to find very small pathogen populations, the probability does not depend on the host population size, or whether it is at a roadside or not. And further, as the unobserved pathogen populations are likely to be small, they also are expected to have minor importance for the landscape-level transmission dynamics.

### Road data

The road network shapefiles, and the Åland archipelago map, visualized in Fig 2, were downloaded from the National Land Survey of Finland as they were in September 2016. Based on the road classification, we omitted pedestrian and cycling roads from our analyses.

### Transmission models

#### Mechanistic transmission model

To gain mechanistic understanding on the transmission process, we fitted an explicit transmission model [32] to the consecutive year-to-year presence-absence records of the pathogen within the local host populations. These indicate the locations of new, persisting and extinct pathogen populations as well as those that remained uninfected during the time-step. This data is informative on the *net transmission events* between populations within a given year. Thus, the true rates of between-population transmission could be higher, as with this data we cannot tell if several transmission events occurred during the summer. Also we neglect the dynamics of local, within-population epidemics, and only consider the observed absolute abundances when defining the model.

The model works in discrete time and we denote with $y_{t}^{i}$ the infection status of population *i* at time *t*, where $y_{t}^{i}=1$ if population is infected and zero othervise. The first modeling assumption is that both the pathogen population extinction rate and the transmission (pathogen emigration) rate of a local pathogen population (i.e. infected host population) depend on the abundance of infection within it. This is justified because pathogen abundance directly corresponds to the amount of spores available for dispersal [33] and correlates with the number of pathogen spores produced for overwintering [34]. We denote with $c(a_{t}^{i})$ the corresponding transmission (or infectiousness) rate parameter for the observed abundance $a_{t}^{i}$ in patch *i* at time *t*, and with $e(a_{t}^{i})$ the probability of infection extinction (at time *t* + 1). We assume that:

$$\begin{array}{c} c\left(a_{t}^{i}\right)=c_{a},\;\text{if}\;\text{infection}\;\text{abundance}\;\text{at}\;\text{population}\;i\;\text{at}\;\text{time}\;t\;\text{was}\;a\text{,}\;a\in \{1,2,3\}, \end{array}$$ (1)

and the same is assumed for $e(a_{t}^{i})$. We thus assume that the infectivity or persistence of an infection do not depend on year, but only on infection abundance. Therefore we assume three infectivity- and pathogen extinction parameters, one for each infection abundance class, that are unknown and thus estimated from the data.

The second modeling assumption is that the dispersal distance of a pathogen spore is distributed according to a negative exponential distribution both along the road and along the land, where we denote with *α*_*road*_ and *α*_*euc*_, the mean dispersal distances of the pathogen spores by roads and by land, respectively. Then, the rate at which any local host population *k* becomes infected during year *t* is defined to be:

$$\begin{array}{c} R_{i}^{t}=\sum_{j,j\neq i}c\left(a_{t}^{j}\right)\times (\theta_{road}\frac{1}{2\pi \alpha_{road}^{2}}e^{-\frac{d_{ij}^{road}}{\alpha_{road}}}+\theta_{euc}\frac{1}{2\pi \alpha_{euc}^{2}}e^{-\frac{d_{ij}^{euc}}{\alpha_{euc}}}), \end{array}$$ (2)

where the terms $\frac{1}{2\pi \alpha^{2}}$ ensure that the dispersal kernel is a probability distribution [35]. Here *θ*_*road*_ and *θ*_*euc*_ are the relative transmission rates along the road and along the land, and $d_{ij}^{road}$ and $d_{ij}^{euc}$ denote the distances between the local populations *i* and *j* along the road network and along the land, respectively (*euc* denoting for Euclidean distance). Distances along the roads were computed by projecting the gravitational centers of the host populations to their closest location within the road network and considering the distances between these projections. Finally, $c(a_{t}^{j})$ corresponds to the transmission rate of the source population *j* at time *t*, and the sum is taken over all the possible source populations. The probability of host population *i* becoming colonized at time *t* is:

$$\begin{array}{c} P\left(y_{t}^{i}=1|y_{t-1}^{i}=0\right)=1-e^{-R_{i}^{t}}. \end{array}$$ (3)

The probabilities of other possible observed transitions in the data are defined as:

$$\begin{array}{ccc} P(y_{t}^{i}=1|y_{t-1}^{i}=1) & = & (1-e(y_{t-1}^{i}))+e\left(y_{t-1}^{i}\right)\times P\left(y_{t}^{i}=1|y_{t-1}^{i}=0\right) \end{array}$$ (4)

$$\begin{array}{ccc} P(y_{t}^{i}=0|y_{t-1}^{i}=1) & = & e\left(y_{t-1}^{i}\right)\times (1-P(y_{t}^{i}=1|y_{t-1}^{i}=0)) \end{array}$$ (5)

$$\begin{array}{ccc} P(y_{t}^{i}=0|y_{t-1}^{i}=0) & = & 1-P(y_{t}^{i}=1|y_{t-1}^{i}=0). \end{array}$$ (6)

#### Model variants

Since it is not known if and how the roads influence the transmission dynamics, we consider three alternative model formulations.

- Model 1: transmission distances are the same, i.e. we assume *α*_*road*_ = *α*_*euc*_, while *θ*_*road*_ and *θ*_*euc*_ are given independent prior distributions.
- Model 2: transmission distances and transmission rates are allowed to be distinct for road- and land-based transmission, i.e. all the parameters *α*_*road*_, *α*_*euc*_, *θ*_*road*_ and *θ*_*euc*_ are all assumed to have independent prior distributions.
- Model 3: transmission rate is assumed to be the same for transmission along the road and along the land, i.e. *θ*_*road*_ = *θ*_*euc*_, while *α*_*road*_ and *α*_*euc*_ are both assumed to be unknown and estimated separately.

#### Inference on mechanistic models using STAN

The target of the inference for the mechanistic transmission model is the joint distribution of the parameters:

$$\begin{array}{c} \psi ≔\{\alpha_{road},\alpha_{euc},\theta_{road},\theta_{euc},c_{1},c_{2},c_{3},e_{1},e_{2},e_{3}\}. \end{array}$$ (7)

We define the likelihood of the parameters as the probability of all the observed within-population transitions: a host population remaining or becoming colonized by the pathogen and the host population becoming or remaining free of infection, as defined in Eqs 3–6.

$$\begin{array}{c} P\left(d|\psi \right)=\prod_{i,t}P\left(y_{t-1}^{i}|y_{t}^{i},\psi \right). \end{array}$$ (8)

As the stationary distribution for the initial states is intractable, we have omitted the term $P(y_{1}^{i},\psi )$, and write the likelihood as a function of the observed transitions in the data. This model is fitted using the probabilistic progamming language STAN [36], sampling 5000 samples from the posterior distribution using variational inference, and the priors for the parameters were set according to Table 1.

**Table 1:** The prior distributions used for the parameters of the mechanistic transmission model. The prior distributions and their truncation were chosen based on inital model fits.

parameter	prior distribution	truncation
*α*_*road*_	normal(1000, 1000)	[1, 10000]
*α*_*euc*_	normal(1000, 1000)	[1, 10000]
*θ*_*road*_	normal(0, 200)	[0.001, 1000]
*θ*_*euc*_	normal(0, 200)	[0.001, 1000]
*c*_*i*_ for *i* ∈ {1, 2, 3}	normal(0, 3000)	[0.001, 10000]
*e*_*i*_ for *i* ∈ {1, 2, 3}	beta(1,1)	[0.001, 10000]

#### Inference on mechanistic models using STAN

Posterior predictive simulations were used for model comparison and evaluation as well as for studying the properties of predicted epidemics. To compare the predictive performances of the three models, we simulated transmission dynamics under each mechanistic model, sampling *ψ* from the corresponding posterior distribution, setting the initial state as in the data at year *t*, and simulating 100 realizations of the presence-absence records for the next year *t* + 1. This was done separately for all the study years *t*, and these simulated observations were then compared to the actual observations. In addition, to illustrate the implications of our results, we performed posterior predictive simulations of explicit epidemics on the landscape using the fitted models and studying the properties of the resulting epidemics. To assess what proportion of all the transmissions occur along the roads as opposed to lands, we initiated 600 random locations to have an infection and then simulated the resulting transmission dynamics over 10 years, keeping track of all the transmissions and their route, e.g. whether they occurred along the road or by the land. As further illustration of the dynamics, we analyzed how under model 2 the starting location of an epidemic influenced its potential to spread within the system. In particular, we seeded epidemics at single locations with high, average and low betweenness and simulated 5000 realizations of 10-year epidemics to assess at what probability the neighbouring locations got infected. In all these simulations we neglected the abundance dynamics, and assumed that each infected pathogen population has abundance level 2, corresponding to 10-100 infected host plants, and remained that way until the infection was cleared.

### Spatio-temporal statistical modeling of locations of pathogen populations

#### Spatiotemporal modeling using network and other covariates with INLA

The mechanistic transmission model only considers the shortest distances between the host populations along the road, omitting thus the information that certain host populations could be connected by the road via several alternative routes, and that the amount of traffic along the roads is not uniform across the network. Therefore, as an alternative analysis, we fitted a generalized additive model on the pathogen presence-absence data, modeling both pathogen presence-absence across the years and the colonization process: i.e. the presence-absence of the pathogen within populations that were found empty the previous year, and using covariates that measure the connectedness and centrality of locations within the road network. To account within the model the possible spatiotemporal dependencies between locations and consecutive years, the model also has a spatiotemporal part, denoted with *z*_*t*_. It is defined by assuming 1st order autoregressive process for the temporal dependency:

$$\begin{array}{c} z_{t}=\rho z_{t-1}+\omega_{t}, \end{array}$$ (9)

*ρ* corresponding to the degree of temporal dependency, and *ω*_*t*_ being a zero-mean Gaussian vector, with spatially structured covariances. We assume *ω*_*t*_ to have Matérn covariance function:

$$\begin{array}{c} C_{i,j}=C\left(d_{i,j},\kappa ,\tau \right)=\frac{\sigma^{2}}{\Gamma \left(\lambda \right)2^{\lambda -1}}{\left(\kappa d_{i,j}\right)}^{\lambda }K_{\lambda }\left(\kappa d_{i,j}\right) \end{array}$$ (10)

where *σ* is the marginal variance and *κ* is a scaling parameter related to the distances at which correlations between locations decay, and these are estimated from the data. Parameter λ is related to the smoothness of the covariance function, which we here set to default value 1. From the fitted spatio-temporal field, the distance at which the correlations have fallen approximately to 0.1, called the *range*, can be fetched: $\frac{{\left(8\lambda \right)}^{1/2}}{\kappa }$. These model structures are similar as in [18] and are further explained in [37], so we refer to those references for exact exposition. Efficient Bayesian inference on such models is possible using R-INLA package [37], which was also utilized here.

#### The covariates for the statistical model

To link each local population to the underlying road network, we calculated for each local population summary statistics based on the location of it relative to road network. For these calculations, the centroid of each local host population was projected and equated with the closest point to it in the road network, and summary statistics and distances to other habitat patches used in the statistical analyses were computed based on these projections. For additional predictors, we used the abundance of infection in the previous year, the local host-coverage and pathogen- and host connectivity, both of which have been previously shown to influence the pathogen dynamics [18]. There was some correlation between the two connectivity measures, but othervise the covariates did not have major correlations between them. The correlations between the covariates and summary statistics of their distribution are given in S1 Fig and in S2 Table.

The first network centrality measure we considered is the *betweenness* [38], that we computed for each host population relative to the road network. In general, for a graph node *v* within a graph *G* is defined by the number of shortest paths going through the node *v*:

$$\begin{array}{c} b\left(v\right)≔\sum_{i,j}\frac{g_{ivj}}{g_{ij}} \end{array}$$ (11)

where *g*_*ivj*_ equals the number of paths traversing from node *i* to node *j* through node *v*, and *g*_*ij*_ is the total number of paths from *i* to *j*. Betweenness is thus high for the hubs of the network, through which many routes are expected to pass, and we assume this also coincides with the amount of traffic passing by.

In addition, we calculated for each host population the *closeness centrality*, that considers the distances to every other node from a given location along the network. In particular, for a vertex *v*, it is defined as the inverse of the sum of the length of the shortest paths between the node *v* and all other nodes in the graph.

$$\begin{array}{c} c\left(v\right)≔\frac{1}{\sum_{i}d\left(v,i\right)}, \end{array}$$ (12)

where *d*(*v*, *i*) is the shortest distance along graph *G* from node *v* to *i*, and the *i* runs over all graph nodes. We computed both summary statistics using the R-package igraph [39].

Previous studies have shown the impact of both host- and pathogen *connectivity* measures on the pathogen epidemiology, and therefore they were included in our modeling. Both measure the expected amount of dispersal into a population from surrounding populations, when exponential dispersal kernel, and no other spatial structure, is assumed. In detail, pathogen connectivity for local population *i* is defined as:

$$\begin{array}{c} S_{i}^{p}=\sum_{j,j\neq i}O_{j}e^{-\frac{d_{i,j}}{\alpha }} \end{array}$$ (13)

where *O*_*j*_ = 1, if population *j* was infected and *O*_*j*_ = 0 othervise. Similarly, the host connectivity is computed as follows:

$$\begin{array}{c} S_{i}^{h}=\sum_{j,j\neq i}\sqrt{A_{j}}e^{-\frac{d_{i,j}}{\alpha }} \end{array}$$ (14)

where *A*_*j*_ is the size (*m*^2^) of the host population *j*. For both connectivity measures, we set *α* = 1000, corresponding to average dispersal distance of 1000 meters, used in other similar studies on the system [18, 40]. The values for *O*_*j*_ and *A*_*j*_ were set based on the observed covariates of the current year. The concept of connectivity is elaborated for instance in [41] and [42]. The resulting measure $S_{i}^{p}$ can be interpreted as the force of infection from the other infected host populations, and similarly $S_{i}^{h}$ measures the expected rate of host immigration to the population *i*, and therefore both statistics can be used as a proxy for the amount of gene flow into the population.

#### Non-linear covariate effects

To allow for non-linear effects of the covariates in the statistical model, the effect of all covariates with continuous support (all other predictors exluding the abundance categories), was modelled by fitting a function of random walk of order 2 to their support:

$$\begin{array}{c} x_{i-1}-2x_{i}+x_{i+1}∼N\left(0,\vartheta^{-1}\right), \end{array}$$ (15)

where *x*_*i*−1_, *x*_*i*_ and *x*_*i*+1_ correspond to consecutive (discretized) values of the considered covariate, and *ϑ* is a parameter describing the smoothness of the resulting estimated function. Uninformative prior distributions for the parameters governing the spatial random field and for the predictors were used [43], also for the smooth effects [44].

#### Model selection

For model selection, we utilize the *Watanabe-Akaike information criterion (WAIC)* [45], that considers the out-of-sample predictive accuracy of the fitted model and corrects for the effective number of parameters within it. We consider several alternative groupings of the above-mentioned predictors and retain the corresponding WAICs to compare the appropriateness of the fitted models.

## Results

### Host populations and road network

**Fig 4:**
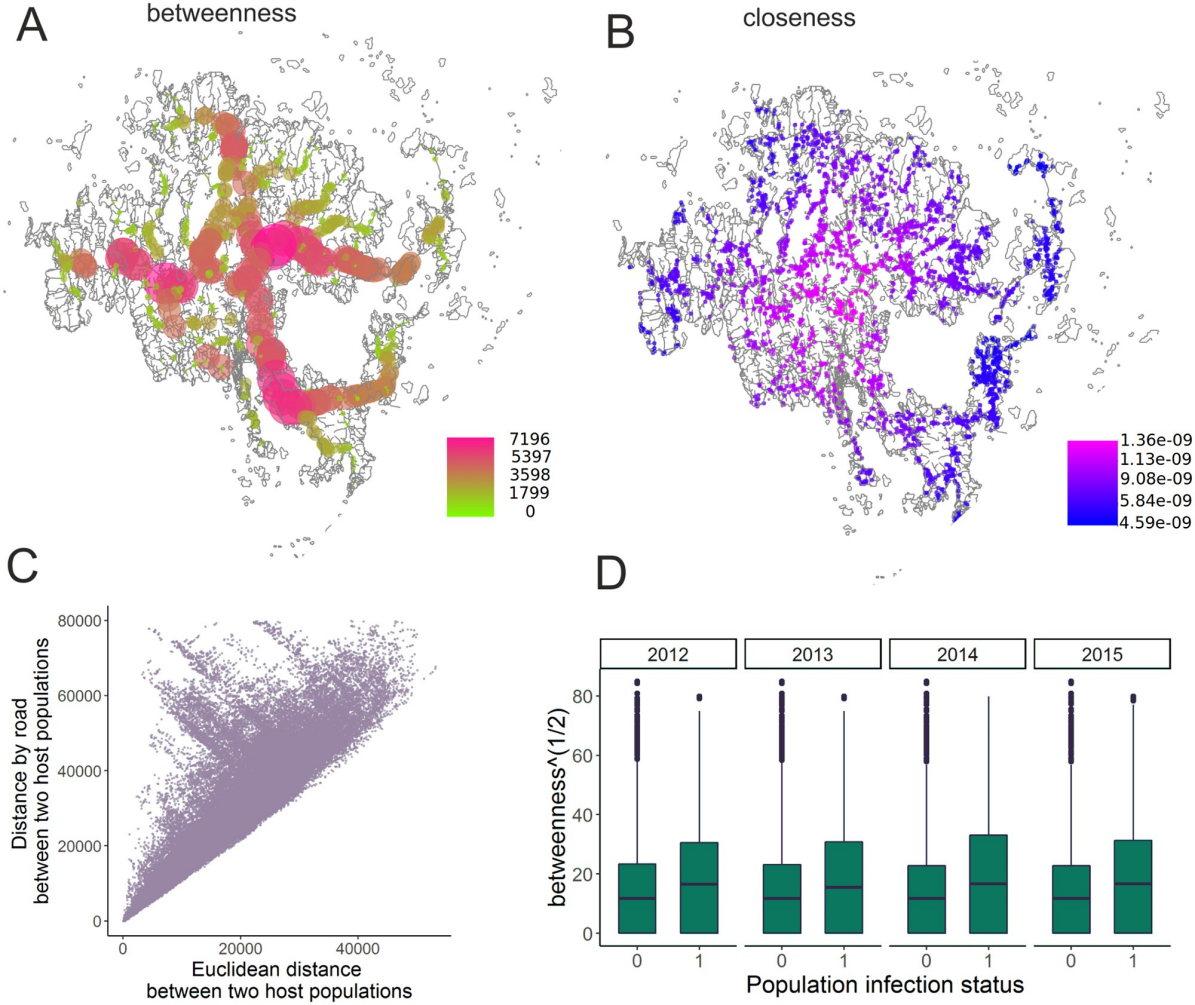
The two computed centrality measures, betweenness (A) and closeness (B), for the considered host populations, computed based on their projection to the closest point in the road network. The correlation between the Euclidean- and shortest distance by road for a random set of pairs of host populations (C) and the relationship between the computed betweenness summary-statistic and the presence and absence of pathogen in different years (D). The roadmaps in the background were created using data produced by National Land Survey of Finland.

As seen in Fig 2, the road network is a planar network with a distinct topology. The computed betweenness and closeness measures are visualized in Fig 4 panels A and B. The large variance in betweenness suggests that the road network deviates from classical grid street plan, and many routes from one place to another pass through the few main roads. The natural consequence of this is that there is often a significant missmatch between the distances by the road and by the land for many pairs of host populations, as seen from panel C in Fig 4. Finally, from Fig 2 panel D, we see that each year the betweenness tends to be higher for the infected populations than for the uninfected ones (the difference in means being statistically significant each year).

### Mechanistic transmission model with road- and land-based transmission

#### Mechanistic model 1

When a different transmission rate was defined for both the land- and road-based transmission, but the dispersal distance distribution was assumed to be the same, the results show that transmission occurs at a much higher rate (posterior mean for *θ*_*road*_ being 151.3 ([121.24, 185.33] 95% CI), and for *θ*_*euc*_ it was estimated to be 19.67 ([5.79, 47.78] 95% CI). The corresponding mean dispersal distance was then estimated to be 404 ([341, 473] 95% CI).

#### Mechanistic model 2

When both the transmission rate and the transmission distance were allowed to differ between roads and land, we acquire similar conclusions, as still the rate of transmission is significantly higher along the roads (posterior mean for *θ*_*road*_ being 148.62 ([112.71, 190.95] 95% CI), and for *θ*_*euc*_ being 30.69 ([7.32, 82.8] 95% CI). The average dispersal distance is inferred to be shorter for road-based transmission than land-based (posterior mean for *α*_*road*_ being 403, ([323, 494] 95% CI), and for *α*_*euc*_ being 1989, ([838, 3643] 95% CI).

#### Mechanistic model 3

When transmission rate is assumed equal regardless of the transmission route, then we infer that the average dispersal distance is shorter for road-based transmission and longer for land-based transmission, posterior mean for *α*_*road*_ being 306 ([240, 383] 95% CI), and for *α*_*euc*_ being 2013 ([772, 3962] 95% CI). The transmission rate parameter *θ* was estimated to have posterior mean 34.01 and [28.07, 40.63] 95% CI.

#### Conclusions on mechanistic models

**Fig 5:**
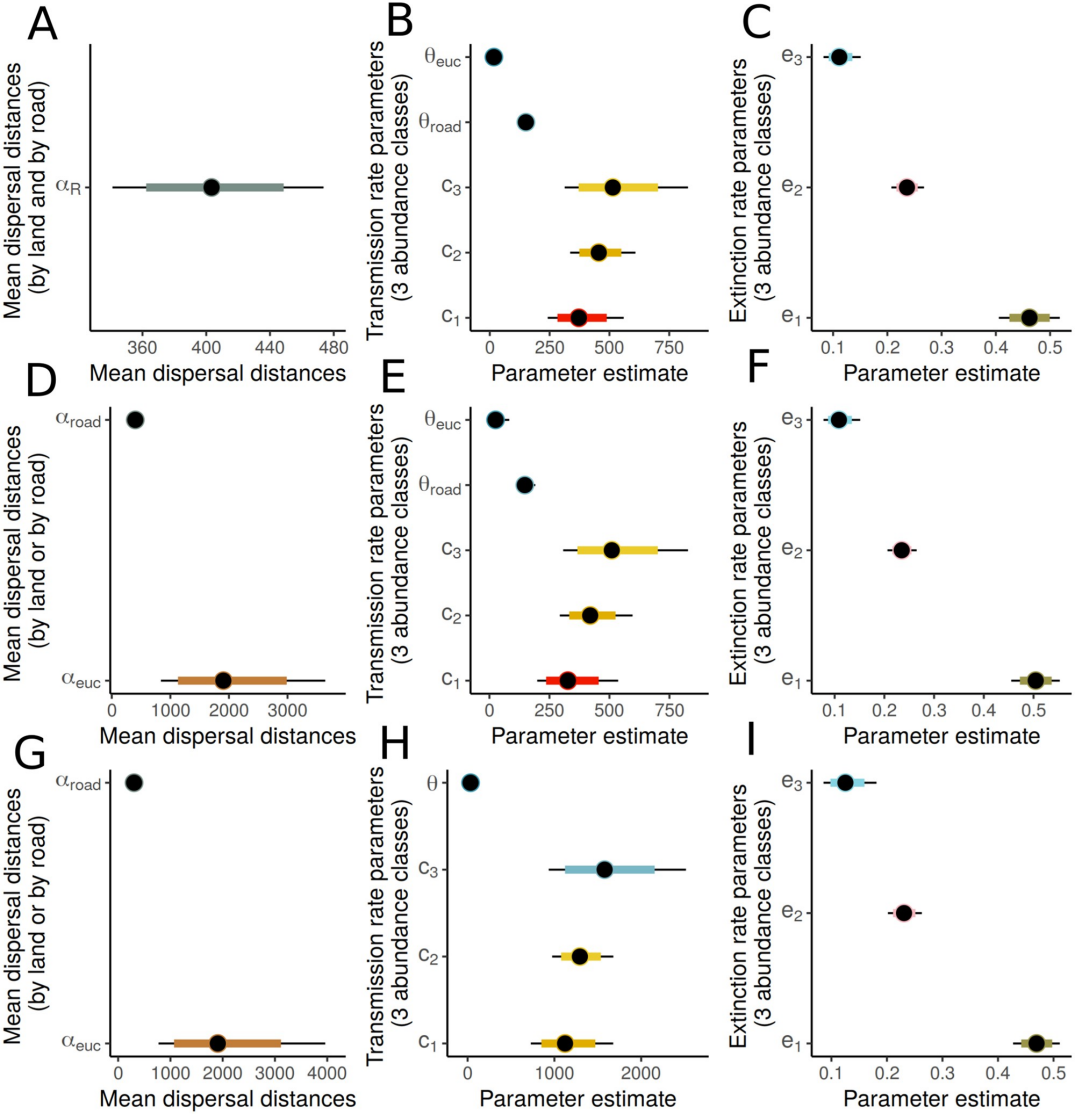
The parameter estimates (medians and 80% and 95% credibility intervals) for the fitted mechanistic within-season transmission models. Panels A, B and C correspond to mechanistic model 1, panels D, E and F correspond to mechanistic model 2 and panels G, H and I correspond to mechanistic model 3. As an example, panel A depicts the estimated mean dispersal distances by land (*α*_*E*_) and by road (*α*_*R*_), while panels B and C depict the estimated colonization rates from patches with different abundance of infection (*c*_1_ being the smallest abundance class), and the estimated pathogen population extinction rates for the pathogen populations with different abundances of infection. The axis limits are different in each plot.

The posterior distributions for the fitted parameters of the different mechanistic transmission models are visualized in Fig 5 and given in exact detail in S1 Table. Overall, the two first models indicate that if *the transmission rate* varies for land- and road-based transmission, it will be considerably higher for road-based transmission. Results from Model 3, in which the transmission rates were assumed to be equal across land and along the roads, suggest that average dispersal distances are shorter along the roads and longer across the land. However based on the estimated confidence intervals, it seems that data is more informative on the mean dispersal distance along the roads, while less so on the mean dispersal distance along the land, leaving some ambiguity on the goodness-of-fit of that model. The predictive performance of all the three models was somewhat similar, and approximately 80% of the predicted events were simulated correctly, yet none of the models was highly accurate in predicting the new transmissions. Best predictive performance was however obtained with model 2, which was driven by its ability to best predict the populations that *remain uninfected*. The predictive success for the different kinds of events for all the three models are shown in S5 Table.

All considered models were structured to allow the observed infection abundances to influence the dispersal- and extinction rates of the different pathogen populations. It is worth noting that the transmission rate parameters in Model 3 have a different interpretation, due to different model structure, and therefore are on a different scale. The results on these parameters across the models suggest that the infection outbound rate is higher when the infection abundance class is higher, while the opposite holds for the pathogen population extinction probability. In particular, the infection outbound rate can be 1/3 larger for abundance class 2 and 1/2 larger for abundance class 3, compared to class 1. For the extinction rates it seems that pathogen populations go extinct with probabilities approximately 0.5, 0.25, and 0.1, when the pathogen abundance previous year was 1, 2 or 3, respectively. Both conclusions match our expectations.

### Road network structure predicts the locations of pathogen populations

**Fig 6:**
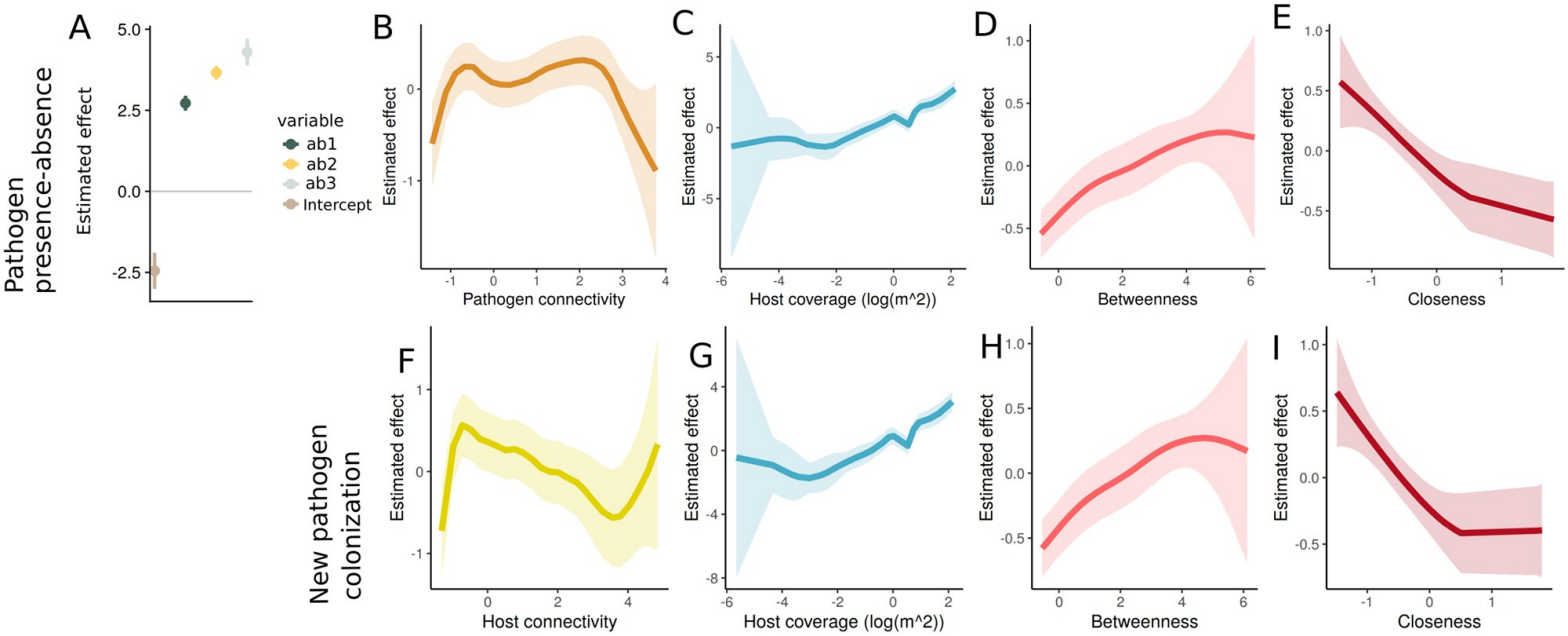
The estimated effects for the predictors for the presence of infection together with the estimated 95% credibility intervals in the first row (panels A, B, C, D and E), and for the model that considers the locations that did not have the pathogen previous year in the second row (panels F, G, H and I). The covariates were scaled prior to analysis and the effects are shown in the scaled axis as well. Panel A depicts the effect of pathogen abundance classes (ab1-ab2) previous year on the infection presence the next year. The results for the models with best WAICs among the considered models are shown.

The results for statistical models with the best predictive accuracy measured by WAIC, are shown in Fig 6, and the results for the corresponding model hyperparameters are shown in Table 2. The WAICs for all the considered statistical models are given in S3 and S4 Tables. When predicting pathogen presence-absence, pathogen abundance in the previous year in the same location has a positive effect (panel A), and there is a positive effect of pathogen connectivity, especially at very low values (panel B). Host coverage within a pathogen population was also associated with a positive effect (panel C). For the road network summary statistics, we find opposing effects of the two network centrality measures: the betweenness is estimated to have a clear positive effect on the pathogen presence (panel E), while closeness has first a negative and then a saturating effect on pathogen presence (panel F). Interestingly, when predicting the new transmissions, i.e. modeling presences and absences considering only locations that did not have the pathogen previous year, pathogen connectivity is not included in the best fitting model. However, we retain the negative effect of host connectivity (panel F), the positive effect of host population size, and similar effects of the road network in this model as in the pure presence-absence model. The estimated hyperparameters both for presence-absence and new colonization events suggest similar spatiotemporal fields, with similar scale nominal variances and spatial ranges (approximately 6km) for both models.

**Table 2:** Posterior medians and 95% credibility intervals for the hyperparameters of the statistical model, where *ρ* describes the 1st order autocorrelation, nominal variance and range describes the spatial random field, where nominal variance describes the overall variance of the field and range corresponds to the distance after which the spatial autocorrelation is estimated to become smaller than 0.1, when the Matern covariance structure is assumed.

Model	*ρ*	Nominal variance	Range (meters)
Presence Absence	0.36, [0.12, 0.56]	0.97, [0.68, 1.38]	6034.9, [4016.8, 9056.9]
New Pathogen population	0.29, [0, 0.54]	0.94, [0.68, 1.31]	6017.7 [4077.9, 8879]

### Simulated epidemics on the landscape

**Fig 7:**
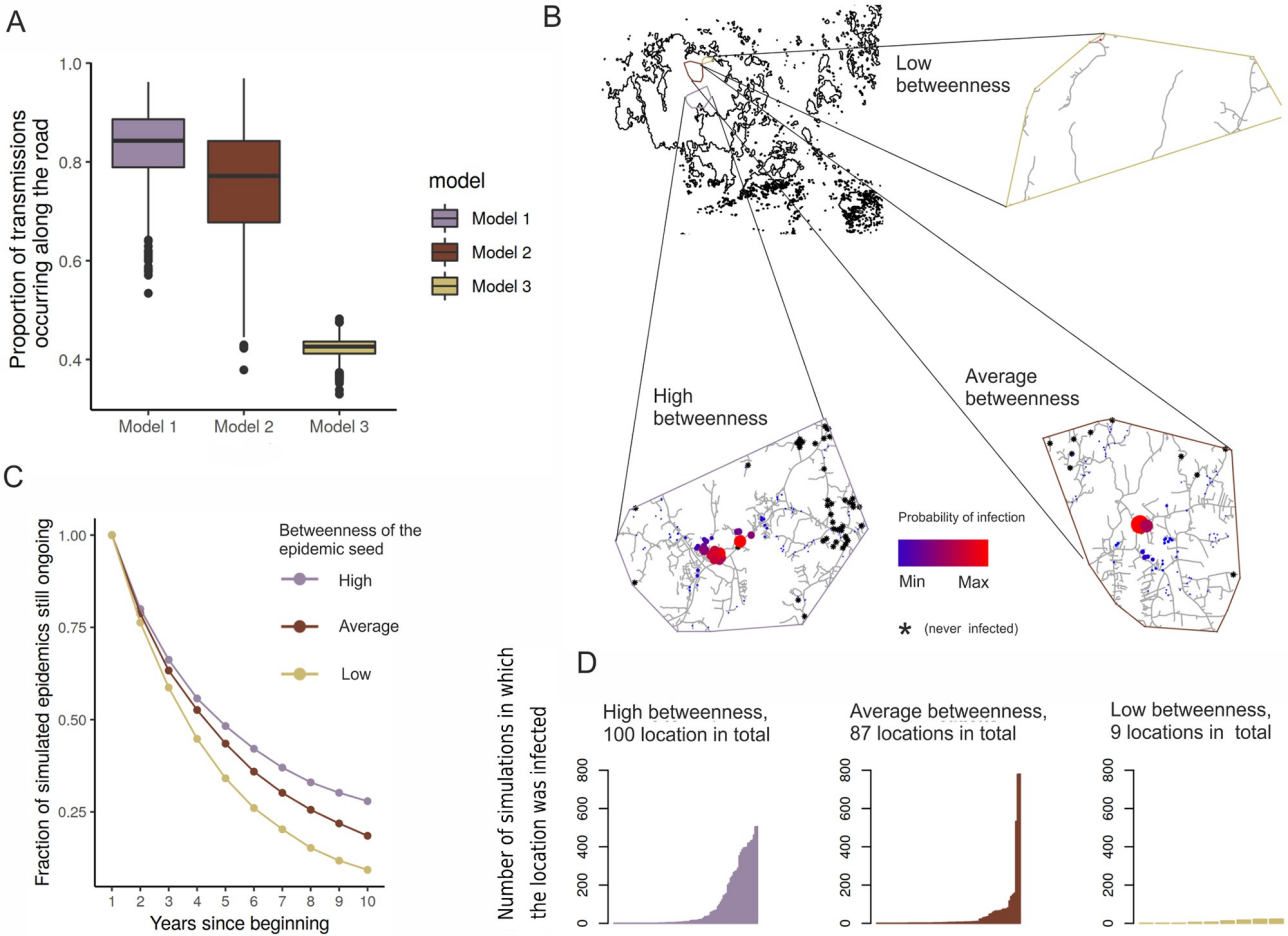
The posterior predictive simulation results with the SIS-models. Panel A shows for each model what proportion of transmissions on average occur via a road-based transmission pathway, and what proportion traverses by land for each three mechanistic transmission models. In panels B-D, we show how the initial location of the epidemic influences its potential to spread (for model 2). In B we have initiated epidemics in locations with high, average and low betweenness, and the colors illustrate in how many simulations (from a total of 5000) the different locations were infected during 10-year’s time. Panel C illustrates for the same epidemic initializations the probability distribution for the epidemic time-span, and D illustrates for the locations that got infected in at least one simulation, in how many simulations in total they were infected. The maps in panel B were created using data produced by National Land Survey of Finland.

In addition to model comparison, posterior predictive simulations allowed us to assess how in practice the epidemics would occur under different modelling assumptions. Based on these simulations, we conclude that the posterior medians for proportion of transmissions that occur along the road are 0.84, 0.77 and 0.43, for Models 1, 2 and 3 respectively. Hence, if different transmission rates are assumed for land- and road transmission, then a considerably larger fraction of transmissions is expected to occur along the roads. If equal rates are assumed, this conclusion does not hold. The boxplots for the simulated proportions are shown in panel A of Fig 7.

For further illustration, we simulated epidemics under Model 2 (that had the best predictive success) with three different kinds of starting locations, and considered high-, average and low betweenness for the initial epidemic locations. In panel B of Fig 7, we show for the neighbouring locations of these focal locations, how likely they are to get infected during a 10-year time interval. Panel C shows the proportion of simulations in which the epidemic spanned a given amount of time, and how this depends on its starting location. We find that an epidemic that started at a very central location has a probability larger than 0.3 to circulate over a 10-year period, while and epidemic started at a low-betweenness location is very likely to have ended. Panel D illustrates the distributions for the distinct neighbouring locations for how often they get infected throughout the 5000 simulations. From this we see, that the epidemics starting from more central locations can yield more varible epidemic outcomes.

## Discussion

In this study we have presented two arguments for showing the significant role of road network and -traffic in the transmission dynamics of the powdery mildew epidemics within the Åland islands archipelago. We further combine these arguments when simulating the transmissions, thereby demonstrating how the road network topology influences the dynamics in the system. This is in agreement with theoretical arguments [46, 47], but to date the empirical support for this has remained scarce. In particular, we show for the first time, that this is not only due to roadsides being a particularly suitable habitat, but due to the roads acting as transmission pathways for the pathogen. The statistical models for infection presence and for new transmissions both indicated that the road-network statistics had a significant effect on the presence of pathogen populations, despite the host abundance within them being accounted for. This was most clearly seen from the estimated effects of the network betweenness, which supposedly has the natural interpretation to coincide with the amount of traffic, as it measures the amount of distinct journeys passing through a location. Estimated negative effect of the closeness suggested that transmissions occur at the fringes of the main island, as opposed to the geographic center. This coincides again with the traffic hypothesis, as the ferry connections to the other islands or to the mainland operate mainly from the south and east. Also the commercial center, Mariehamn is situated at a bay at very south of the main island. By mechanistic modeling of the transmission process, we took a step towards a more realistic understanding of the transmission process of *P. plantaginis*. Our results suggest that if transmission is assumed to occur at a different rate along the roads than across the land, it truly is more frequent along the roads. This coincides with the hypothesis that the wind gusts from bypassing traffic cause turbulence that facilites the inital take-off of the spores. It may also lead to stepping stone-style dispesal along the roads, caused by consecutive take-offs and landings of the spores. The predictive checks with the mechanistic models however indicate uncertainty especially when predicting new transmissions, suggesting that the mechanistic models are potentially missing some relevant information, such as the amount of traffic along the different roads.

Previous studies on the same pathosystem have mostly focused on estimating the mean dispersal distance along a landscape that was considered homogenenous [18, 40], neglecting the possibility of several different transmission pathways and the possibility of differential transmission rates along these pathways. While the estimated mean dispersal distances here differ slightly from those estimated without the road network included, they are similar in magnitude (our estimates being 300-2000 meters depending on the route, vs. the previous estimate of 860 meters in [40]). However the results we present here from the three different mechanistic transmission models also illustrate that the estimated dispersal distance highly depends on the assumptions made on the dispersal rate and dispersal pathways. This suggests that in general the interpretation of the estimated parameters is not unambiguous, and that such estimates might not be transferrable to another landscape. Our study thus brings up two methodological questions related to modeling disease spread, or species dispersal in general, within complex landscapes. First, when dispersal is assumed to occur through several pathways, their unique contributions need to be carefully assessed, as they might not be strongly identifiable from each other and the resulting posterior distributions could be multimodal. Similar identifiablity issues could occur when trying to disentangle the actual rate of dispersal from the dispersal distance distribution, as was seen in our study. A second methodological challenge would involve development of spatial models, e.g. Gaussian processes for networks, that could quantify the flow of information through all possible pathways along the network, and not just through the shortest route.

In conclusion, we highlight the strong influence of the human handprint, here the road traffic, on disease dynamics within a semi-natural landscape. We expect that similar considerations would be needed to correctly understand the transmission in other agricultural- and wild disease systems, or any ecological system with complex dispersal processes. Apart from traffic networks, we believe that a network-based analysis may be necessary, when studying for instance ecological systems within river networks [21], when dispersal occurs through ocean currents [48], or when dealing with established animal migration routes [47].

## Supporting information

S1 Fig Correlations between different covariates in the statistical model. (PDF)

S1 Table Summaries of the posterior distributions, means, standard deviations and posterior quantiles, for the estimated SIS-transmission model. (PDF)

S2 Table Summary statistics for the covariates used in the statistical model. (PDF)

S3 Table The computed WAICs for presence-absence models with different predictors. (PDF)

S4 Table The computed WAICs for new pathogen population models with different predictors. (PDF)

S5 Table The predictive performance of the three mechanistic models. (PDF)
