# Supplementary material for: The spread of a wild plant pathogen is driven by the road network
Source: PLoS Comput Biol. 2020 Mar 31;16(3):e1007703. doi: 10.1371/journal.pcbi.1007703 (PMC7108725; doi:10.1371/journal.pcbi.1007703)
Supplement: S1 Fig — (PDF) [file pcbi.1007703.s001.pdf]

Supporting information ”The spread of a wild plant pathogen is driven by the road network”

Elina Numminen* & Anna-Liisa Laine
* elina.numminen@helsinki.fi

S1 Figure

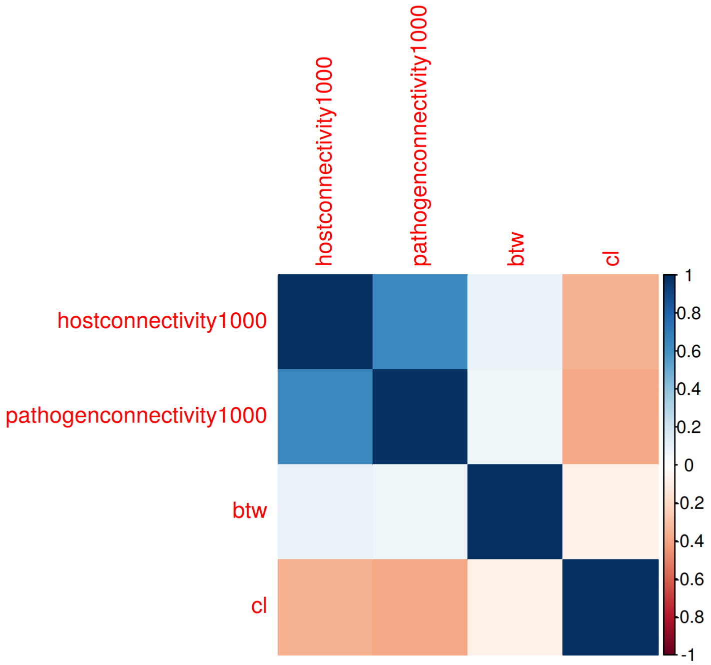

Fig 1. Correlations between different covariates in the statistical model: the two connectivity measures and the the two network summary statistics betweenness (btw) and closeness (cl).
