# Supplementary material for: The spread of a wild plant pathogen is driven by the road network
Source: PLoS Comput Biol. 2020 Mar 31;16(3):e1007703. doi: 10.1371/journal.pcbi.1007703 (PMC7108725; doi:10.1371/journal.pcbi.1007703)
Supplement: S1 Table — (PDF) [file pcbi.1007703.s002.pdf]

Supporting information "The spread of a wild plant pathogen is driven by the road network"

Elina Numminen* & Anna-Liisa Laine
* elina.numminen@helsinki.fi

S1 Table

parameter	model	mean	sd	q2.5.	q50.	q97.5.
α_{road}	Model 1	404.47	33.80	341.16	403.38	473.59
θ_{road}	Model 1	151.30	16.15	121.24	150.91	185.33
θ_{euc}	Model 1	19.67	11.28	5.79	17.09	47.78
c_1	Model 1	380.62	81.68	242.07	372.21	559.65
c_2	Model 1	459.75	69.80	335.90	455.47	608.70
c_3	Model 1	528.40	130.81	313.02	514.12	828.33
e_1	Model 1	0.46	0.03	0.41	0.46	0.52
e_2	Model 1	0.24	0.02	0.21	0.24	0.27
e_3	Model 1	0.11	0.02	0.08	0.11	0.15
α_{road}	Model 2	403.95	43.65	323.40	401.57	494.78
α_{euc}	Model 2	1989.19	732.55	838.88	1905.61	3643.87
θ_{road}	Model 2	148.62	20.12	112.71	147.44	190.95
θ_{euc}	Model 2	30.69	20.13	7.32	25.56	82.80
c_1	Model 2	338.39	87.92	199.17	326.88	536.39
c_2	Model 2	426.05	77.43	293.39	419.76	596.30
c_3	Model 2	524.33	134.14	307.33	509.79	827.24
e_1	Model 2	0.50	0.03	0.46	0.50	0.55
e_2	Model 2	0.24	0.01	0.21	0.23	0.26
e_3	Model 2	0.11	0.02	0.08	0.11	0.15
α_{road}	Model 3	306.89	36.65	240.20	304.75	383.08
α_{euc}	Model 3	2013.02	810.64	772.79	1906.74	3962.22
c_1	Model 3	1146.00	243.90	728.39	1122.63	1677.28
c_2	Model 3	1302.07	180.24	974.32	1293.39	1678.75
c_3	Model 3	1614.79	401.03	934.33	1578.64	2515.97
θ	Model 3	34.01	3.22	28.07	33.88	40.63
e_1	Model 3	0.47	0.02	0.43	0.47	0.51
e_2	Model 3	0.23	0.02	0.20	0.23	0.26
e_3	Model 3	0.13	0.02	0.09	0.13	0.18

Table 1. Summaries of the posterior distributions, means, standard deviations and posterior quantiles, for the three estimated SIS-transmission models.
