# Supplementary material for: The spread of a wild plant pathogen is driven by the road network
Source: PLoS Comput Biol. 2020 Mar 31;16(3):e1007703. doi: 10.1371/journal.pcbi.1007703 (PMC7108725; doi:10.1371/journal.pcbi.1007703)
Supplement: S2 Table — (PDF) [file pcbi.1007703.s003.pdf]

Supporting information ”The spread of a wild plant pathogen is driven by the road network”

Elina Numminen* & Anna-Liisa Laine
* elina.numminen@helsinki.fi

S2 Table

Variable	min	1st Quantile	Mean	3rd quantile	Max
Host connectivity	0	16.77	32.92	43.67	158.62
Pathogen connectictivity	0	1.78	3.55	4.7	13.93
Betweenness	0	0	1502186	342851	51794071
Closeness	4.382e-10	6.963e-10	1.005e-09	1.071e-09	1.839e-09
Host coverage	0.000	0.4	4.85	4	80

Table 1. Summary statistics for the observed values of the covariates used in the statistical model.
