# Supplementary material for: The spread of a wild plant pathogen is driven by the road network
Source: PLoS Comput Biol. 2020 Mar 31;16(3):e1007703. doi: 10.1371/journal.pcbi.1007703 (PMC7108725; doi:10.1371/journal.pcbi.1007703)
Supplement: S3 Table — (PDF) [file pcbi.1007703.s004.pdf]

Supporting information ”The spread of a wild plant pathogen is driven by the road network”

Elina Numminen* & Anna-Liisa Laine
* elina.numminen@helsinki.fi

S3 Table

Model	Abundance	Pathogen connectivity	Host Connectivity	Betweenness	Closeness	Host coverage	WAIC
Model 1	x	x	x	x	x	x	7954.24
Model 2	x	x	x	x		x	7978.72
Model 3	x	x	x		x	x	8011.68
Model 4	x	x		x	x	x	7919.86
Model 5	x		x	x	x	x	7932.27
Model 6	x			x	x	x	7938.95
Model 7	x	x	x			x	8021.96
Model 8	x			x		x	7951.90
Model 9	x				x	x	7974.06
Model 10	x	x				x	7980.15
Model 11	x		x			x	7970.00
Model 12	x					x	7989.35

Table 1. The computed WAICs for presence-absence models with different predictors, ’x’ denoting that the covariate was included in the model.
