# Supplementary material for: The spread of a wild plant pathogen is driven by the road network
Source: PLoS Comput Biol. 2020 Mar 31;16(3):e1007703. doi: 10.1371/journal.pcbi.1007703 (PMC7108725; doi:10.1371/journal.pcbi.1007703)
Supplement: S4 Table — (PDF) [file pcbi.1007703.s005.pdf]

Supporting information ”The spread of a wild plant pathogen is driven by the road network”

Elina Numminen* & Anna-Liisa Laine
* elina.numminen@helsinki.fi

S4 Table

Model	Pathogen connectivity	Host Connectivity	Betweenness	Closeness	Host coverage	WAIC
Model 1	x	x	x	x	x	6046.238
Model 2	x	x	x		x	6048.083
Model 3	x	x		x	x	6075.146
Model 4	x		x	x	x	6021.640
Model 5		x	x	x	x	6005.024
Model 6			x	x	x	6019.132
Model 7	x	x			x	6075.399
Model 8			x		x	6029.349
Model 9				x	x	6048.682
Model 10	x				x	6056.769
Model 11		x			x	6033.100
Model 12					x	6060.645

Table 1. The computed WAICs for new pathogen population models with different predictors, ’x’ denoting that the covariate was included in the model.
