# Supplementary material for: The spread of a wild plant pathogen is driven by the road network
Source: PLoS Comput Biol. 2020 Mar 31;16(3):e1007703. doi: 10.1371/journal.pcbi.1007703 (PMC7108725; doi:10.1371/journal.pcbi.1007703)
Supplement: S5 Table — (PDF) [file pcbi.1007703.s006.pdf]

Supporting information "The spread of a wild plant pathogen is driven by the road network"

Elina Numminen* & Anna-Liisa Laine
* elina.numminen@helsinki.fi

S5 Table

From the total of 1392600 events predicted, the numbers of correct predictions were: 1215828, 1219588 and 1214012, for models 1, 2, and 3, respectively.

The transitions in the table are defined as:
1 = Becomes colonized by pathogen
2 = Ends the colonization of the pathogen
3 = Remains colonized
4 = Remains uncolonized

Model	True Transitions	Predicted transitions 1	2	3	4
Model 3	1	2502	0	0	68698
	2	0	16725	33775	0
	3	0	40458	117242	0
	4	33841	0	0	1079359

Model	True Transitions	1	2	3	4
Model 2	1	2231	0	0	68969
	2	0	17926	32574	0
	3	0	41557	116143	0
	4	29912	0	0	1083288

Model	True Transitions	1	2	3	4
Model 3	1	2645	0	0	68555
	2	0	16883	33617	0
	3	0	40316	117384	0
	4	36100	0	0	1077100

Table 1. The prediction success for the different mechanistic models 1, 2, and 3. The true transition observed in the data is shown in the rows, whereas the columns indicate the predictions from the posterior.
